# Supplementary material for: Towards the prediction of non-peptidic epitopes
Source: PLoS Comput Biol. 2022 Feb 18;18(2):e1009151. doi: 10.1371/journal.pcbi.1009151 (PMC8893639; doi:10.1371/journal.pcbi.1009151)
Supplement: S1 Appendix — Fig A. Distribution of the non-peptidic epitopes within each cluster. Table A. Ontology enrichment analysis of all ChEBI clusters. (DOCX) [file pcbi.1009151.s001.docx]

# **S1 Appendix. Full results of PCA and BiNChE analyses**


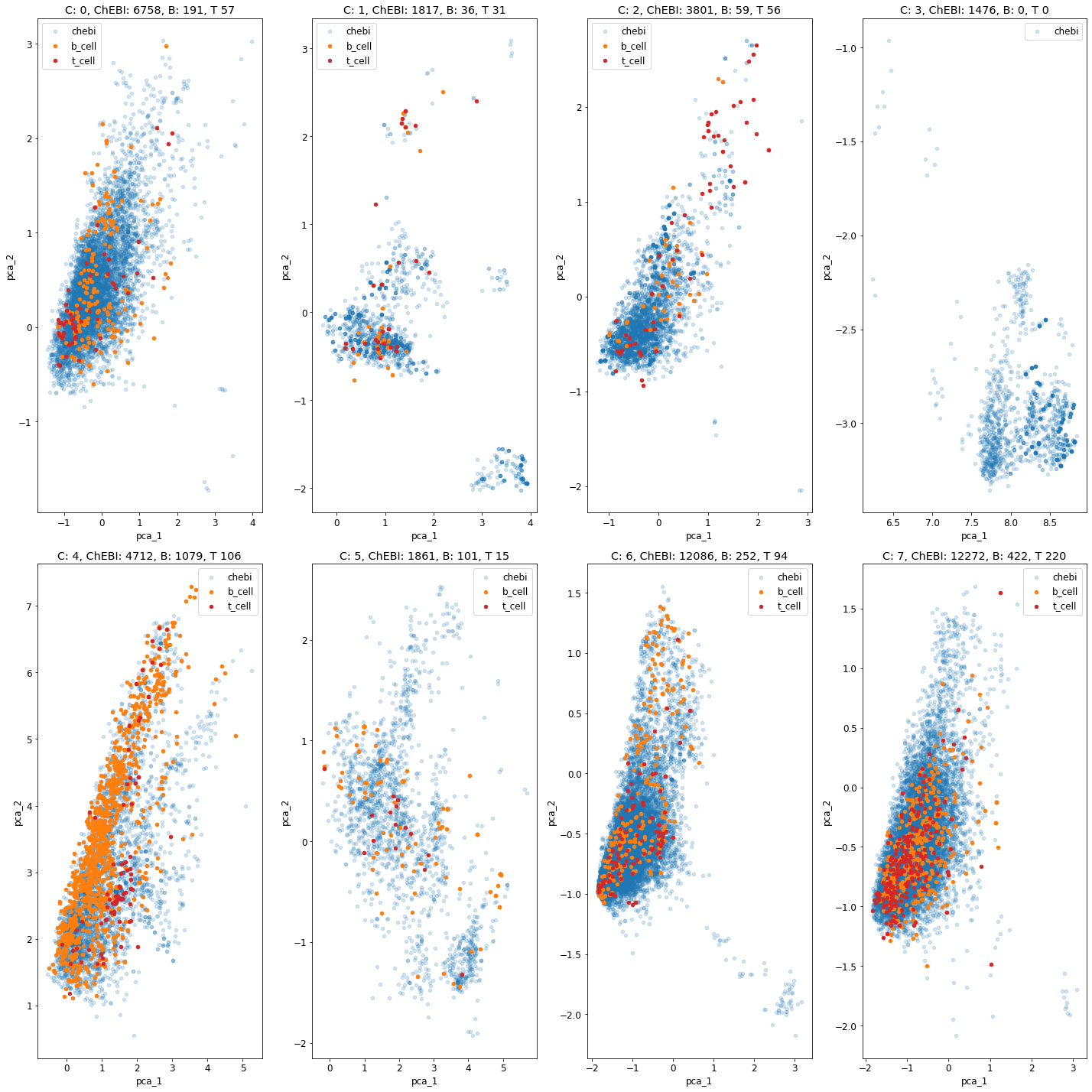


**Fig A.** Distribution of the non-peptidic epitopes within each cluster.

**Table A.** Ontology enrichment analysis of all ChEBI clusters.

| **Cluster** | **ChEBI ID** | **ChEBI Name** | **Fold-**  **enrichment** | **Sample (%)** |
| --- | --- | --- | --- | --- |
| 0 | CHEBI:33595 | cyclic compound | 1.36 | 0.67 |
| 0 | CHEBI:25367 | molecule | 1.23 | 0.73 |
| 0 | CHEBI:33635 | polycyclic compound | 2.79 | 0.52 |
| 0 | CHEBI:33832 | organic cyclic compound | 1.35 | 0.66 |
| 0 | CHEBI:72695 | organic molecule | 1.22 | 0.73 |
| 0 | CHEBI:36963 | organooxygen compound | 1.27 | 0.64 |
| 0 | CHEBI:25806 | oxygen molecular entity | 1.37 | 0.79 |
| 0 | CHEBI:33304 | chalcogen molecular entity | 1.28 | 0.80 |
| 0 | CHEBI:26878 | tertiary alcohol | 11.31 | 0.08 |
| 0 | CHEBI:30879 | alcohol | 6.84 | 0.22 |
| 1 | CHEBI:37739 | glycerophospholipid | 18.03 | 0.43 |
| 1 | CHEBI:35741 | glycerolipid | 15.92 | 0.55 |
| 1 | CHEBI:18059 | lipid | 4.54 | 0.66 |
| 1 | CHEBI:16247 | phospholipid | 16.31 | 0.50 |
| 1 | CHEBI:37734 | phosphoric ester | 9.3 | 0.51 |
| 1 | CHEBI:35701 | ester | 5.26 | 0.65 |
| 1 | CHEBI:26079 | phosphoric acid derivative | 8.23 | 0.67 |
| 1 | CHEBI:26082 | phosphorus molecular entity | 7.94 | 0.67 |
| 1 | CHEBI:33241 | oxoacid derivative | 5.68 | 0.67 |
| 1 | CHEBI:37577 | heteroatomic molecular entity | 1.86 | 0.68 |
| 2 | CHEBI:25806 | oxygen molecular entity | 1.53 | 0.88 |
| 2 | CHEBI:33304 | chalcogen molecular entity | 1.41 | 0.88 |
| 2 | CHEBI:36586 | carbonyl compound | 2.59 | 0.47 |
| 2 | CHEBI:35748 | fatty acid ester | 8.49 | 0.05 |
| 2 | CHEBI:18059 | lipid | 4.52 | 0.66 |
| 2 | CHEBI:78840 | olefinic compound | 3.9 | 0.12 |
| 2 | CHEBI:25384 | monocarboxylic acid | 8.24 | 0.30 |
| 2 | CHEBI:33575 | carboxylic acid | 4.42 | 0.32 |
| 2 | CHEBI:64709 | organic acid | 4.29 | 0.33 |
| 2 | CHEBI:24833 | oxoacid | 4.32 | 0.32 |
| 3 | CHEBI:61902 | hydroxy fatty acyl-CoA | 74.3 | 0.08 |
| 3 | CHEBI:62618 | hydroxyacyl-CoA | 73.99 | 0.11 |
| 3 | CHEBI:17984 | acyl-CoA | 70.45 | 0.51 |
| 3 | CHEBI:37240 | adenosine 3',5'-bisphosphate | 70.37 | 0.51 |
| 3 | CHEBI:37123 | nucleoside bisphosphate | 68.69 | 0.51 |
| 3 | CHEBI:25608 | nucleoside phosphate | 36.89 | 0.51 |
| 3 | CHEBI:33302 | pnictogen molecular entity | 1.93 | 0.99 |
| 3 | CHEBI:33833 | heteroarene | 7.52 | 0.51 |
| 3 | CHEBI:33659 | organic aromatic compound | 2.1 | 0.51 |
| 3 | CHEBI:37734 | phosphoric ester | 9.28 | 0.51 |
| 4 | CHEBI:36233 | disaccharide | 16.01 | 0.03 |
| 4 | CHEBI:50699 | oligosaccharide | 14.24 | 0.07 |
| 4 | CHEBI:16646 | carbohydrate | 6.49 | 0.08 |
| 4 | CHEBI:78616 | carbohydrates and carbohydrate derivatives | 7 | 0.77 |
| 4 | CHEBI:36963 | organooxygen compound | 1.55 | 0.78 |
| 4 | CHEBI:25806 | oxygen molecular entity | 1.45 | 0.84 |
| 4 | CHEBI:33304 | chalcogen molecular entity | 1.34 | 0.84 |
| 4 | CHEBI:36962 | organochalcogen compound | 1.42 | 0.78 |
| 4 | CHEBI:63567 | tetrasaccharide derivative | 22.77 | 0.05 |
| 4 | CHEBI:63563 | oligosaccharide derivative | 21.27 | 0.27 |
| 5 | CHEBI:7754 | oligonucleotide | 53.29 | 0.05 |
| 5 | CHEBI:61120 | nucleobase-containing molecular entity | 22.37 | 0.48 |
| 5 | CHEBI:33302 | pnictogen molecular entity | 1.61 | 0.83 |
| 5 | CHEBI:33833 | heteroarene | 7.5 | 0.51 |
| 5 | CHEBI:33659 | organic aromatic compound | 2.13 | 0.52 |
| 5 | CHEBI:25608 | nucleoside phosphate | 15.25 | 0.21 |
| 5 | CHEBI:37734 | phosphoric ester | 4.62 | 0.25 |
| 5 | CHEBI:26079 | phosphoric acid derivative | 6.34 | 0.52 |
| 5 | CHEBI:36359 | phosphorus oxoacid derivative | 6.29 | 0.52 |
| 5 | CHEBI:36360 | phosphorus oxoacids and derivatives | 6.21 | 0.52 |
| 6 | CHEBI:37577 | heteroatomic molecular entity | 1.51 | 0.55 |
| 6 | CHEBI:33240 | coordination entity | 4.2 | 0.03 |
| 6 | CHEBI:33676 | d-block molecular entity | 4.52 | 0.04 |
| 6 | CHEBI:37022 | amino-acid anion | 6.96 | 0.02 |
| 6 | CHEBI:29067 | carboxylic acid anion | 2.62 | 0.09 |
| 6 | CHEBI:25696 | organic anion | 2.15 | 0.15 |
| 6 | CHEBI:22563 | anion | 2.38 | 0.18 |
| 6 | CHEBI:25699 | organic ion | 1.79 | 0.17 |
| 6 | CHEBI:35406 | oxoanion | 2.78 | 0.11 |
| 6 | CHEBI:25741 | oxide | 2.81 | 0.12 |
| 7 | CHEBI:33832 | organic cyclic compound | 1.27 | 0.62 |
| 7 | CHEBI:33595 | cyclic compound | 1.27 | 0.63 |
| 7 | CHEBI:50047 | organic amino compound | 2.41 | 0.15 |
| 7 | CHEBI:33659 | organic aromatic compound | 1.99 | 0.48 |
| 7 | CHEBI:36683 | organochlorine compound | 4.55 | 0.08 |
| 7 | CHEBI:36684 | organohalogen compound | 3.58 | 0.13 |
| 7 | CHEBI:37578 | halide | 3.91 | 0.17 |
| 7 | CHEBI:24471 | halogen molecular entity | 3.81 | 0.17 |
| 7 | CHEBI:37577 | heteroatomic molecular entity | 1.64 | 0.60 |
| 7 | CHEBI:50995 | secondary amino compound | 6.91 | 0.03 |
